# MM-BMSCs induce naïve CD4+ T lymphocytes dysfunction through fibroblast activation protein α

**DOI:** 10.18632/oncotarget.17538

**Published:** 2017-04-30

**Authors:** Xiaofei Wu, Yadan Wang, Jian Xu, Ting Luo, Jun Deng, Yu Hu

**Affiliations:** ^1^ Department of Hematology, Institute of Hematology, Union Hospital Tongji Medical College Huazhong University of Science and Technology Wuhan, Hubei, 430022 China

**Keywords:** multiple myeloma, bone marrow mesenchymal stromal cells, fibroblast activation protein α, immunosuppression, CD4+ T cells

## Abstract

**Background:**

The tumor microenvironment plays a major role in multiple myelomas (MM). MM-BMSCs (bone marrow mesenchymal stromal cells) can support tumor growth and immune surveillance escape. On the other hand, fibroblast activation protein α, expressed by cancer stroma cells including BMSCs, has been shown to potentiate epithelial cancers growth and immune suppression.

**Results:**

MM-BMSC inhibited proliferation of T cells (*P* = 0.0138), promoted senescence of T cells (*P* < 0.001), consistent with decreased CD28 and hTERT expression (*P* < 0.001), Treg/Th17 was down-regulated by MM-BMSC (*P* = 0.031). After treatment with FAPα inhibitor PT-100, senescent rate was decreased (*P* = 0.001), Treg/Th17 was up-regulated (*P* = 0.024). FAPα was up-regulated by TCCM (*P* = 0.02). p-AKT was increased in MM-BMSC co-cultured T cells (*P* = 0.021) and decreased by PT-100 (*P* = 0.017). Higher level of TGF-β was observed in MM-BMSC co-cultured medium (*P* < 0.001), and down-regulated by PT-100 (*P* = 0.038). p-AKT was upregulated as compared to T-cells without MM-BMSCs (*P* = 0.021). The abnormal p-AKT level was distinctly decreased by PT-100 (*P* = 0.017).

**Materials and Methods:**

The expression of FAPα was analyzed by western blot and RT-PCR. The proliferation and senescence of CD4+ T cells was examined by cck-8 and β-gal staining, and Treg/Th17, CD28 expression was analyzed by FCM. The FAPα and PI3K pathway was analyzed by western blot and their relationship with T cell function was detected by FCM and RT-PCR. The level of IL-10, IL-17 and TGF-β was detected by ELISA.

**Conclusions:**

MM-BMSCs inhibit T-cell proliferation and drive Th17 differentiation through FAPα/TGF-β axis, leading to the progression of myeloma. FAPα-induced T-cell senescence is mediated by the PI3K signaling pathway.

## INTRODUCTION

Multiple myeloma (MM) is a clonal B-cell malignancy characterized by an accumulation of clonal plasma cells in the bone marrow (BM), leading to bone destruction and BM failure. MM patients typically present with humoral immunodeficiency, which was mainly due to abnormalities in B cells [1]. MM cells and cells in the bone marrow, such as mesenchymal stem cells (MSCs), osteoblasts, osteoclasts, endothelial cells, immunocytes (DC and macrophages), and adipocytes constitute a complex tumor microenvironment that supports growth and immune escape of MM cells [2]. Normal MSCs have been shown to be highly immunosuppressive, suppressing T-cell proliferation, and cytokine production, contributing to the differentiation to CD4+CD25+Foxp3+ T lymphocytes [3–5]. This suppressive effect is supported by direct MSCs-T cell contact, cytokines, and chemokines produced by MSCs, such as TGF-β, PGE2, IDO, nitric oxide, indoleamine 2,3-dioxygenase, and IL-10, which partially favor these adverse effects [5–8]. A previous study demonstrated that the immunomodulatory functions of bone marrow mesenchymal stromal cells (BMSCs), derived from MM patients, are impaired. A reduced inhibition of T lymphocyte proliferation, a lower ability to silence mitogen-stimulated T-cells in G0/G1 phase, a reduced inhibition of T-cell activation, a reduced rate of T-cell apoptosis and increased Th17/Treg ratio have been observed during the co-culture of CD4^+^T-cells with MM-BMSCs compared to the co-cultures with healthy donor (HD) BMSCs [9].

The fibroblast activation protein α (FAPα) is known to be associated with immunosuppression in the tumor. FAPα is a member of serine protease family possessing gelatinase activity and type I collagenase activity, which is a vital type II transmembrane protein expressed in more than 90% epithelial tumor stromal cells [10]. FAPα is also expressed in the membrane of BMSCs and another kind of MSCs [11], and FAPα expressed by stromal cells in tumor microenvironment promote MM cells and glioblastoma growth [12, 13]. High FAP expression is associated with tumor re-growth, recurrence, and poor clinical outcome in pancreatic cancer [14]. Recently, Kraman et al. reported that FAPα-expressing cells were involved in tumor immunosuppression [15]. FAPα-positive cells in tumor microenvironment can produce high levels of inhibitory factors such as TGF-β, IL-6, and IL-10 [16]. The depletion of FAPα-positive cells in LL2 Lewis lung cancer mice rescued the immune response against tumor [15]. MM-BMSCs act as a protectant for bortezomib-induced apoptosis in MM cells through FAPα/β-catenin pathway [12]; however, the other tumor promoting effect, such as immunosuppression, has not yet been fully elucidated.

Several studies found that the accumulation of senescent T-cells in certain types of cancers indicate that it might be utilized by tumor cells to escape immune surveillance [17, 18]. Senescent T-cells are characterized by the loss of CD27 and CD28 expression, cell cycle arrest, and telomere shortening [17, 19]. These senescent T-cells can suppress antigen nonspecific and allogeneic-induced proliferation of normal T-cells. The senescent T-cells expressing few CD25, FoxP3, and CTLA4, possess defective killing abilities and development of potent suppressive activity, leading to dysregulation of the immune system [19–21]. Furthermore, tumor cells can induce senescent T-cells *in vitro* [19, 21], consistent with increased CD28^−^senescent T-cells in cancer patients, which are shown to be immunosuppressive. Senescent T cells secrete large amounts of suppressive cytokines, such as IL-6, IL-10, and TGF-β1; IL-10 and TGF-β1 play crucial roles in the maintenance of an immunosuppressive environment [20]. The phosphatidylinositol 3-kinase (PI3K) pathway is involved in human cell senescence by AKT and mTOR. The inappropriate activation of PI3K pathway drives aging-associated changes in NF-κB function in CD4+ T-cells, and mTOR inhibitor rapamycin reduces the transcriptional activity of NF-κB in senescent CD4+ T-cells [22]. Intracellular reactive oxygen species (ROS) level of systematic lupus erythematosus (SLE) BMSCs is increased in response to activation of PI3K/AKT/FoxO3 signaling pathway, leading to the senescent phenotype of BMSCs [23].

In the present study, we investigated proliferation, apoptosis, differentiation, and senescence of naïve CD4+ T-cells co-cultured with BMSCs and found that MM-BMSCs induced CD4+ T-cell senescence and Th17 differentiation. We further examined the immune functions of CD4^+^ T-cells co-cultured with MM-BMSC after treatment with FAPα inhibitor PT-100 and determined the role of FAPα in immunosuppression. In addition, our study revealed that FAPα expressed by MM-BMSCs exerted an immunosuppressive effect through TGF-β and PI3K signaling pathways.

## RESULTS

### Identification of BMSCs

BMSCs are fibroblast-like cells with adherence properties and potential to differentiate into adipoblasts, osteoblasts, and chondroblasts. Cultured MSCs express CD105 (SH2), CD73 (SH3/4), andCD90 (Thy-1), but are negative for the expression of hematopoietic markers, such as CD34, CD45, CD14 or CD11b, CD19 or CD79, and HLA-II. In the current study, cultured BMMSCs were adherent to the flasks, exhibited fibroblast-like morphology, were positive for CD90 and CD105, and negative for CD34 and CD45 (Supplementary Figure 1).

### MM-BMSCs exert immunosuppressive effect on T-cells

#### MM-BMSC inhibit the proliferation of CD4+ T-cells

**Figure 1:**
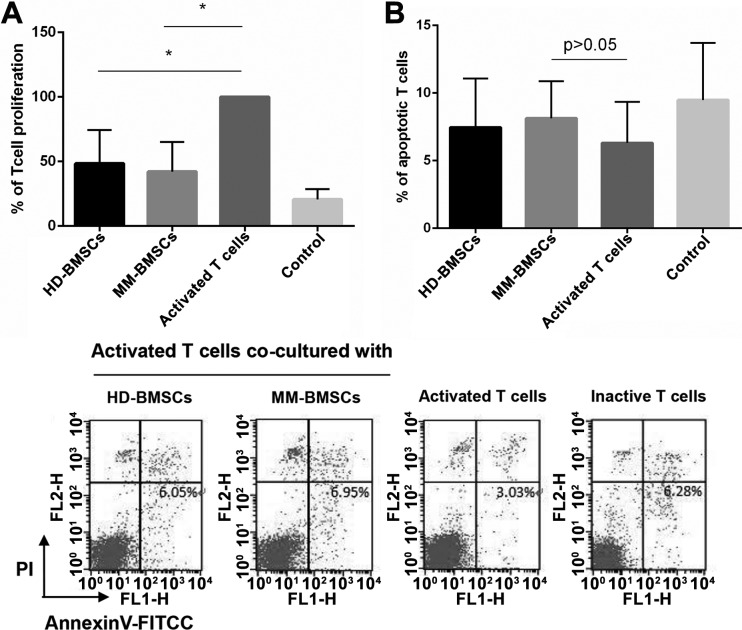
The effect of BMSCs on CD4+ T-cells proliferation and apoptosis (**A**) Percentage of CD4+ T-cells proliferation activated by anti-CD3 functional Ab and IL-2 (50 μg/mL) in co-cultured HD-/MM-BMSCs (5:1) and inactive T cells compared to a 100% T-cell proliferation without BMSCs. All cells were detected by CCK-8 on day 6 (*n* = 9). (**B**) The percentage of apoptotic cells in activated T-cells co-cultured with or without HD-/MM-BMSCs and that of inactive T cells; apoptotic cells were AnnexinV-FITC-positive (low right area) and detected by FCM (*n* = 10).

A previous study demonstrated that BMSCs apparently suppress the proliferation of T-cells, and the inhibitory effect of MM-BMSCs on T cells was impaired compared to HD-BMSCs [9]. We evaluated the proliferation of CD4+ T-cells in the presence and absence of MM-BMSCs and HD-BMSC by CCK-8 kit. Both HD-BMSCs and MM-BMSCs distinctly inhibited the proliferation of CD4+ T-cells activated by anti-CD3 Ab (*P* = 0.0239, 0.0138, respectively). However, no significant difference was observed in the proliferation activity between MM-BMSCs and HD-BMSCs groups (Figure 1A).

### BMSCs did not show obvious inhibitory effect on apoptosis of CD4+ T-cells

In a previous study, activation-induced apoptosis of T-cells increased in a co-culture system in the presence of healthy MSCs, which was demonstrated by increased Annexin V-positive cells and increased expression of cleaved Caspase 3 protein; however, for MM-MSCs, this apoptosis promoting effect was severely impaired [24]. In our study, following stimulation by the anti-CD3 functional antibody, the rate of apoptotic T-cells was 6.31 ± 3.0%. The rate increased slightly when co-cultured with HD-BMSCs or MM-BMSCs, 7.46 ± 3.6% or 8.73 ± 2.7% respectively, BMSCs did not significantly affect the apoptosis of CD4+ T-cells (Figure 1B, *P* > 0.05).

### MM-BMSC promote the senescence of CD4+ T-cells

**Figure 2:**
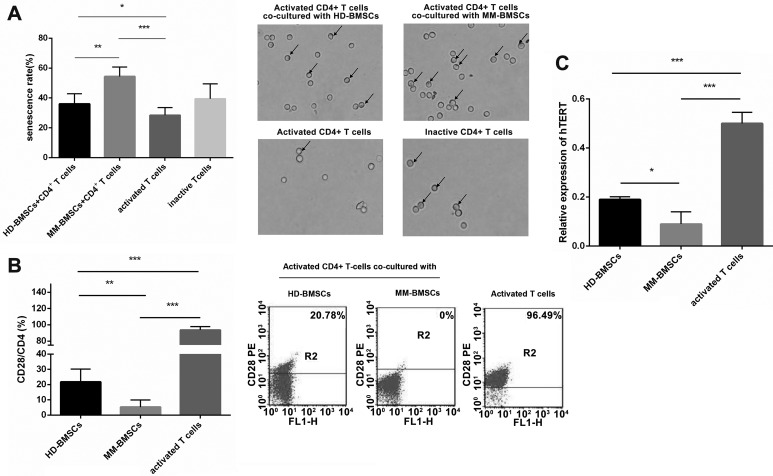
Role of BMSCs in senescence of CD4+ T-cells (**A**) Percentage of CD4+ T-cells senescence activated by anti-CD3 functional Ab and IL-2 (50 μg/mL) co-cultured with HD-/MM-BMSCs (5:1) and inactive T cells. All the senescent cells were detected by β-gal kit on day 3 (*n* = 10). Blue-dyed cells were considered as senescent T-cells. (**B**) CD28 expression on activated CD4+ T-cells treated with or without HD-/MM-BMSCs (*n* = 3). T-cells in upper panel were considered expressing CD28 (*n* = 3). *P < 0.05, **P < 0.01, ***P < 0.001.

BMSCs in the tumor microenvironment are known to exert a suppressive effect on T-cells; for example, inhibiting proliferation, activation, and cytotoxicity [9, 24]. However, the influence of BMSCs on the senescence of T-cells is yet ill-understood. SA-β-gal was the first biomarker used to identify senescent human cells. The senescence of CD4+ T-cells co-cultured with HD-BMSCs or MM-BMSCs assessed by SA-β-gal assay kit showed that both cells facilitated the senescence of CD4+ T-cells (*P* = 0.042, *P* < 0.001, respectively). Compared to HD-BMSCs, MM-BMSCs significantly increased the percentage of SA-β-gal-positive senescent T-cells (Figure 2A). Senescent T-cells are characterized by the loss of CD27 and CD28 expressions and exhibiting negative immune function [17–19]. As shown in Figure 2B, although HD-BMSCs decreased the CD28 expression on CD4+ T-cells (*P* < 0.001, compared to activated T-cells), MM-BMSCs downregulated the expression of co-stimulatory molecules CD28 dramatically (*P* = 0.004, compared to HD-BMSC group, *P* < 0.001, compared to activated T-cells), suggesting that MM-BMSCs induced immune dysfunction in T-cells. The importance of telomerase activity in regulating the process of replicative senescence has been documented via stable *hTERT* (human telomerase reverse transcriptase) gene transduction in fibroblasts, endothelial cells, and T cells, as it is a telomerase catalytic subunit. A noticeable decrease was seen in the relative transcription level of hTERT when CD4+ T lymphocytes were co-cultured with HD-BMSCs and MM-BMSCs (Figure 2C, *P* < 0.001). Consecutively, the expression of hTERT was distinctly downregulated by MM-BMSCs compared to HD-BMSCs (Figure 2B, *P* = 0.011), revealing that telomerase activity was downregulated by MM-BMSCs dramatically.

### MM-BMSCs promoted naive CD4+ T-cells differentiation to Th17

**Figure 3:**
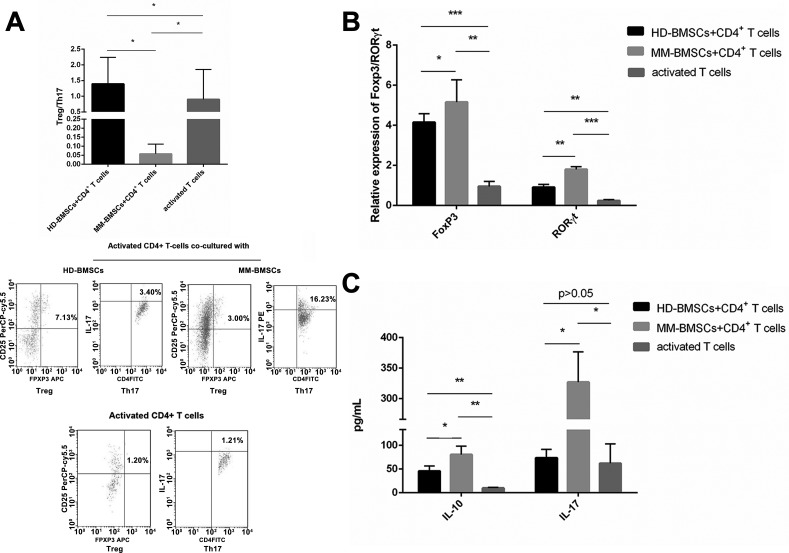
The role of BMSCs in the differentiation of CD4+ T-cells (**A**) The proportion of Treg and Th17 in CD4+ T-cells activated by CD3 Ab and IL-2, co-cultured with or without HD-/MM-BMSCs, assessed by FCM (*n* = 5). The results were demonstrated in Treg/Th17. (**B**) The relative expression of FoxP3 and RORγt in CD4+ T-cells activated by CD3 Ab and IL-2, co-cultured with or without HD-/MM-BMSCs, detected by RT-PCR (*n* = 9). (**C**) The level of IL-10 and IL-17 in the supernatant of co-cultured medium and activated TCCM detected by ELISA (*n* = 3). *P < 0.05, **P < 0.01, ***P < 0.001.

The suppressive effect of HD-BMSCs was associated with an increased percentage of CD4+CD25+Foxp3+ regulatory T-cells and IL-10 secretion [5]. In MM, the ratio of Th17/Treg in peripheral blood is elevated as compared to healthy individuals, and MM-BMSCs lead to a shift of Treg/Th17 balance to Th17 [9, 25]. In our study, HD-BMSCs promoted the induction of Treg, resulting in an upregulated ratio of Treg/Th17 (Figure 3A, *P* = 0.031). However, CD4+ T-cells co-cultured with MM-BMSCs were stimulated to Th17 differentiation (Figure 3A, *P* = 0.03), consistent with an elevated level of IL-17 (Figure 3C, *P* = 0.021). We also found both HD-BMSCs and MM-BMSCs induced the secretion of IL-10 (*P* = 0.002, 0.003, respectively); however, the level of IL-17 was apparently higher than IL-10 in MM-BMSCs co-cultured supernatant (*P* = 0.022). The Th17-specific and Treg-specific transcription factors are the orphan nuclear receptor RORγt and FoxP3. As shown in Figure 3B, MM-BMSCs elevated the expression of RORγt and FoxP3 (*P* < 0.001, *P* = 0.001, respectively). HD-BMSCs mainly affect the expression of FoxP3, leading to differentiation to Treg; however, RORλt- and the IL-17-inducing effect is overwhelming in MM-BMSCs.

### Expression of FAPα in BMSCs from MM patients is induced in MM microenvironment

**Figure 4:**
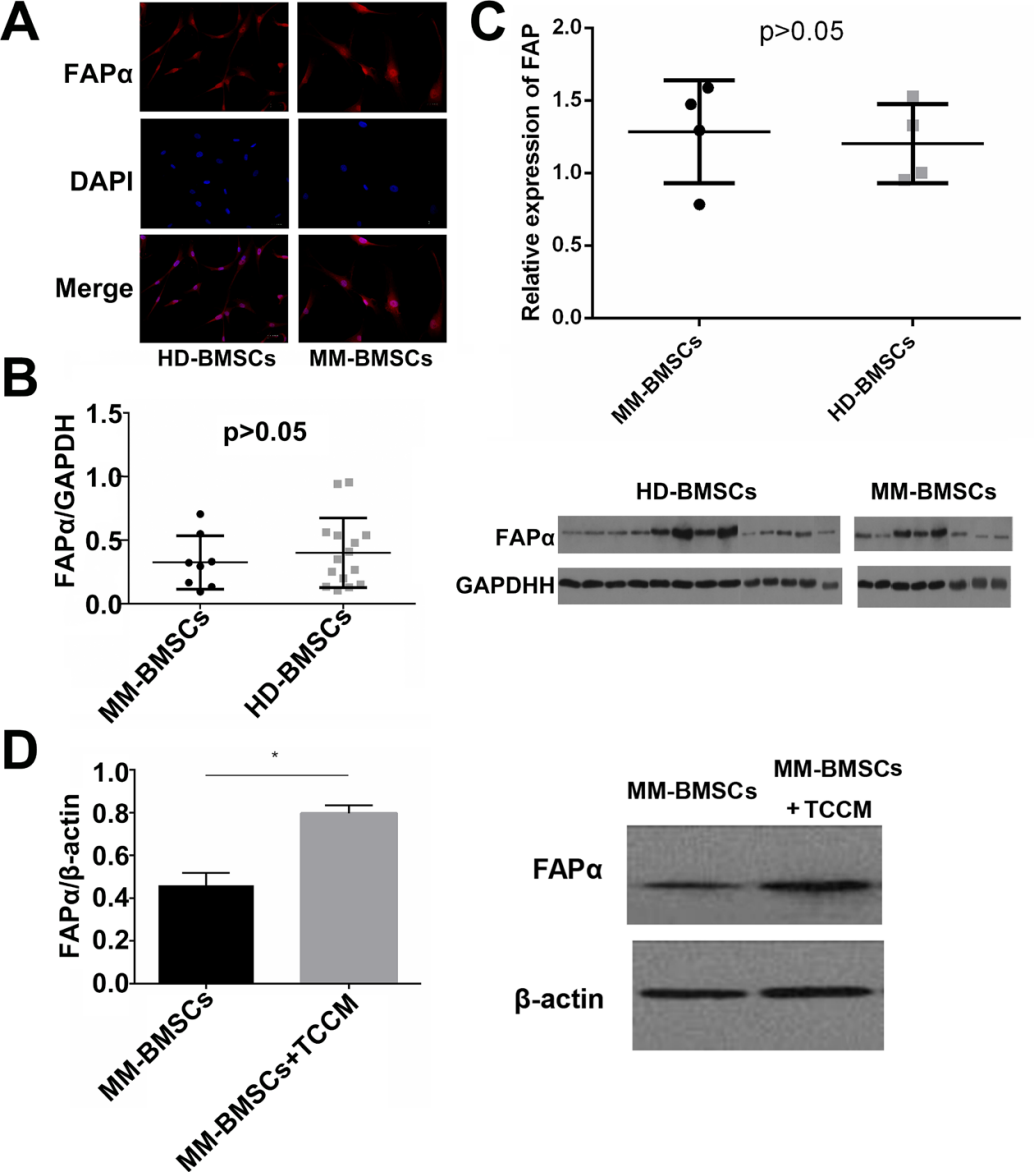
Expression of FAPα on BMSCs (**A**) The expression of FAPα on HD-BMSCs and MM-BMSCs detected by immunofluorescence. (**B**) FAPα expression in HD-BMSCs (*n* = 15) and MM-BMSCs (*n* = 8) detected by Western blot. The results were demonstrated as FAPα/GAPDH. (**C**) The expression of FAP in HD-BMSCs and MM-BMSCs detected by RT-PCR (*n* = 3). (**D**) The expression of FAP in MM-BMSCs with or without TCCM detected by Western blot (*n* = 3). *P < 0.05.

We first detected the expression of FAPα by Western blot and immunofluorescence. Both HD-BMSCs and MM-BMSCs expressed FAPα on the cell membrane (Figure 4A). As shown in Figure 4B, FAPα/GAPDH of HD-BMSCs and MM-BMSCs were 0.40 ± 0.27 and 0.32 ± 0.21 respectively, no significant difference was observed between MM patients and healthy donors (*P* > 0.05). A similar result was observed by RT-PCR (Figure 4C) (*P* > 0.05). To create a similar tumor microenvironment, we added tumor cells cultured medium (TCCM) from MM cells to BMSC-cultured medium (1:1), and the expression of FAPα was evaluated again. As seen in Figure 4D, FAPα/β-actin value was increased from 0.46 ± 0.057 to 0.80 ± 0.038, the protein expression of FAPα was increased when MM-BMSCs were stimulated by TCCM (*P* = 0.02).

### Inhibiting FAPα efficiently upregulated the proliferation of CD4+ T-cells

**Figure 5:**
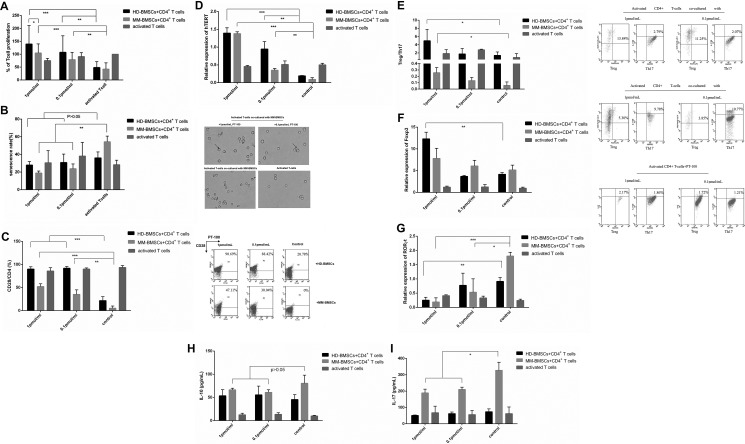
MM-BMSCs regulated CD4+ T-cells through FAPα (**A**) Percentage of CD4+ T-cells proliferation in co-culture with HD-/MM-BMSCs (5:1), which were treated with or without 1 pmol/mL and 0.1 pmol/mL. All groups were assessed by CCK-8 on day 6 and compared to a 100% T-cell proliferation without BMSCs (*n* = 9). (**B**) Percentage of CD4+ T-cells senescence in co-culture with HD-/MM-BMSCs (5:1), which were treated with or without 1 pmol/mL and 0.1 pmol/mL. All cells were detected by β-gal kit on day 3 (*n* = 7). (**C**) The expression of CD28 on activated T-cells in co-culture with HD-/MM-BMSCs, which were treated with or without PT-100 as compared to activated T-cells cultured alone. All cells were assessed by FCM (*n* = 3). (**D**) Relative expression of hTERT in co-culture with HD-/MM-BMSCs, which were treated with or without PT-100, analyzed by RT-PCR (*n* = 5). (**E**) The proportion of Treg and Th17 in CD4+ T-cells activated by CD3 Ab and IL-2, in co-culture with or without HD-/MM-BMSCs, tested by FCM (*n* = 5). The results were demonstrated in Treg/Th17. (**F**) The relative expression of FoxP3 and (**G**) RORγt in CD4+ T-cells activated by CD3 Ab and IL-2, in co-culture with or without HD-/MM-BMSCs, detected by RT-PCR (*n* = 9). (**H**) The level of IL-10 and (**I**) IL-17 in the supernatant of co-culture medium and activated T-cells culture medium assessed by ELISA (*n* = 3). *P < 0.05, **P < 0.01, ***P < 0.001.

CD4+ T-cells was co-cultured with MM-and HD-BMSCs (ratio = 5:1) in the presence or absence of 1pmol/mL and 0.1 pmol/mL PT-100. After 6 days, the proliferative activity of CD4+ T-cells was detected by the CCK-8 kit. Inhibiting FAPα increased the proliferation of T-cells both in MM- and HD-BMSCs co-culture system (Figure 5A, *P* < 0.01); however, the effect is not concentration-dependent.

### Inhibiting FAPα retarded the senescence of CD4+ T-cells

CD4+ T-cells were co-cultured with MM-and HD-BMSCs at the same ratio in presence or absence of 1 pmol/mL and 0.1 pmol/mL PT-100. On day 3, we collected and dyed the senescent T-cells by the β-gal kit, and the degree of senescence was evaluated by the percentage of blue-dyed T-cells. PT-100 efficiently reduced the percentage of blue- dyed cells in CD4+ T-cells co-cultured with MM-BMSCs (Figure 5B, *P* = 0.001). However, PT-100 did not influence the senescence of activated T-cells and the T-cells co-cultured with HD-BMSCs (*P* > 0.05). We found that the expression of CD28 in MM-BMSCs-induced senescent CD4+ T-cells was restored markedly after treatment of 1 pmol/mL and 0.1 pmol/mL PT-100 (Figure 5C, *P* < 0.001, *P* = 0.002, respectively). As expected, 1 pmol/mL and 0.1 pmol/mL PT-100 also stimulated the expression of hTERT in CD4+ T-cells co-cultured with MM-BMSCs (Figure 5D, *P* < 0.001, *P* = 0.001, respectively), suggesting a vital role of FAPα plays in the senescence of immune system.

### PT-100 reversed the Th17 inducing effect of MM-BMSCs

Inhibiting FAPα promoted the Treg/Th17 balance shift to Treg. The ratio of Treg/Th17 in MM-BMSCs co-cultured CD4+ T-cells was upregulated from 0.06 ± 0.05 to 0.26 ± 0.08 (*P* = 0.024), while PT-100 did not affect the Treg/Th17 balance in CD4+ T-cell alone (Figure 5E, *P* > 0.05). The activity of RORγt in the CD4+ T-cells co-cultured with MM-BMSCs was reduced by PT-100 (Figure 5G, *P* < 0.001, *P* = 0.035, respectively), in agreement with declined IL-17 production (Figure 5I, *P* = 0.024). On the other hand, no difference was seen in Foxp3 and IL-10 expression of T-cells co-cultured with MM-BMSCs (Figure 5F and 5H, *P* > 0.05). PT-100 was able to reverse the ratio of Treg/Th17 to Treg, which might interdict the suppressive effect of Th17, suggesting that FAPα plays a major role in MM-BMSCs-mediated immunosuppressive effect.

### FAPα is associated with overexpression of p-AKT

**Figure 6:**
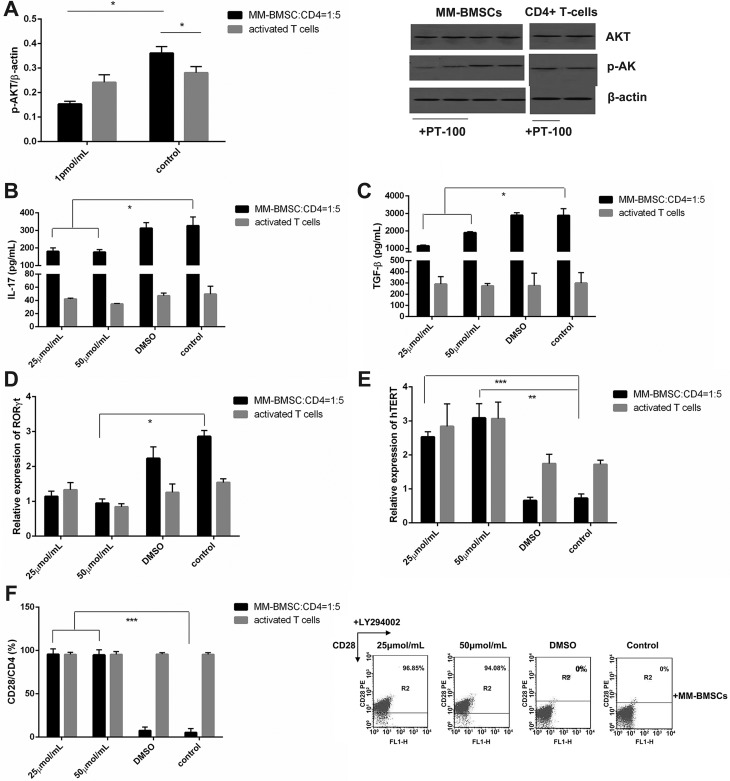
The immunosuppressive effect of FAPα is associated with inappropriate activation of the PI3K signaling pathway (**A**) The level of AKT and p-AKT in activated CD4+ T-cells co-cultured with MM-BMSCs compared to T-cells cultured without MM-BMSCs, detected by Western blot (*n* = 3). The results were demonstrated by p-AKT/β-actin. (**B**) The level of IL-17 and (**C**) TGF-β in the supernatant of MM-BMSCs and CD4+ T-cells co-cultured medium and activated T-cells cultured medium with or without LY 294002, detected by ELISA (*n* = 3). (**D**) RORγt and (**E**) hTERT expression in activated CD4+ T-cells co-cultured with or without MM-BMSCs, evaluated by RT-PCR (*n* = 5). These cells were treated with LY294002 or DMSO. (**F**) CD28 co-stimulatory molecule expression on CD4+ T-cells co-cultured with MM-BMSCs (*n* = 3). These cells were treated with LY294002 or DMSO. *P < 0.05, **P < 0.01, ***P < 0.001.

To gain an insight into the molecular mechanism underlying the FAPα-mediated negative regulation of T-cell function, we measured AKT and p-AKT from CD4+ T-cells in the presence or absence of MM-BMSCs by the Western blot. As shown in Figure 6A, p-AKT/β-actin value in MM-BMSC co-cultured T-cells and activated T-cells were 0.361 ± 0.027 and 0.242 ± 0.031 respectively, p-AKT was upregulated as compared to T-cells without MM-BMSCs (*P* = 0.021). To further elucidate the relationship between elevated FAPα and precocious PI3K activation, we used PT-100 to inhibit the FAPα-mediated function of MM-BMSCs. The abnormal p-AKT level was distinctly decreased to 0.153 ± 0.012 (Figure 6A, *P* = 0.017), Owing to the inappropriate PI3K activation in MM-BMSCs co-cultured CD4+ T-cells, we can conclude that MM-BMSCs might suppress CD4+ T-cell function through the FAPα/PI3K pathway.

### AKT inhibitor, LY294002, rescued abnormal T-cell differentiation and senescence

To our knowledge, appropriate PI3K stimulation drives CD4+ T-cell senescence from older individuals and might be associated with BMSCs senescence. We further proposed that MM-BMSCs induce the T-cell dysfunction through FAPα/PI3K pathway. We used 25 μmol/L and 50 μmol/L LY294002 to inhibit the AKT signaling pathway. As seen in Figure 6B, the IL-17 level was reduced in CD4+ T-cells co-cultured with MM-BMSCs when treated with LY294002 (*P* = 0.032, 0.017, respectively), which is consistent with a reduced Th17-defining transcriptional factor, RORγt (Figure 6D, *P* = 0.034). The data in Figure 6E and 6F documented an increase in hTERT with elevated CD28 expression (*P* < 0.001) after LY294002 treatment, indicating PI3K activation, especially that AKT is associated with T-cell senescence. Furthermore, LY294002 downregulated the TGF-β secretion (Figure 6C, *P* = 0.045, 0.024, respectively), which may also contribute to decreased Th17 ratio and IL-17 production.

### Abnormal expression of cytokines in MM microenvironment

**Figure 7:**
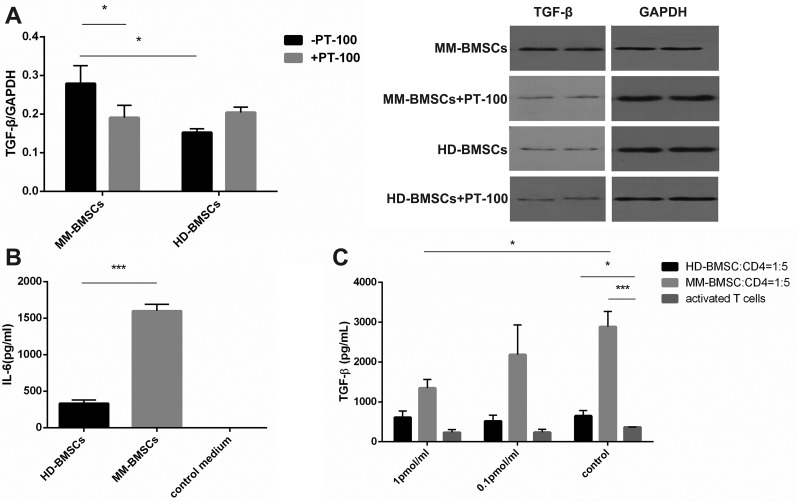
Abnormal cytokine expression in MM-BMSCs (**A**) The expression of TGF-β in MM-BMSCs with or without PT-100 detected by Western blot (*n* = 4). (**B**) The level of IL-6 secretion in HD-BMSCs- and MM-BMSCs-cultured medium analyzed by ELISA (*n* = 5). The fresh BMSCs culture medium was used as a control. (**C**) The level of TGF-β secretion in activated T-cells co-cultured or solitary cultured medium treated with or without PT-100, assessed by ELISA (*n* = 3). *P < 0.05, ***P < 0.001.

The level of IL-6 was increased in MM-BMSCs-cultured medium, as assessed by ELISA (Figure 7B, *P* < 0.001). MM-BMSCs induces IL-6 secretion, which activates the NF-κB pathway, leading to the upregulation of adhesion factors between MM-BMSCs and plasma cells. TGF-β was moderately elevated in MM-BMSCs, as detected by Western blot, and 1 pmol/mL PT-100 downregulated the expression of TGF-β (Figure 7A, *P* = 0.048). A dose-dependent decrease in TGF-β was also observed, indicating that FAPα might mediate the immunosuppressive effect of MM-BMSCs by TGF-β. Thus, TGF-β could suppress the T-cell response [26]. IL-6 and TGF-β play a pivotal role in regulating the balance between Treg and Th17, thereby FAPα may induce immunosuppression through IL-6 and TGF-β.

## DISCUSSION

The present study aimed to improve the understanding of the immunosuppressive function exerted by MM-BMSCs on T-cells and the development of effective strategies to reverse the suppressive tumor microenvironment. Our study focused on the immunoregulation of MM-BMSCS on immune senescence and CD4+ T-cell differentiation. We further demonstrated that MM-BMSCs-induced immune suppression is mediated by FAPα, and TGF-β is responsible for the suppressive effect of FAPα. In addition, we also identified the role of MM-BMSCs in human tumor-mediated immune suppression in order to provide a novel strategy to reverse the immunosuppressive effects of MM microenvironments for tumor immunotherapy.

HD-BMSCs possess the inhibitory effect of T-cell proliferation and induce apoptosis [3, 4, 27] while the inhibitory effect and apoptosis- stimulating effect of MM-BMSCs is impaired notably [9]. The proliferative assay demonstrated that the restraining proliferative activity was independent of the sources of BMSCs. Some previous studies found co-cultured T-cells from MM patients with MM-BMSCs, characterized by senescent phenotype, and silenced in the G0/G1 phase. However, we isolated normal T-cells from healthy donors and co-cultured with MM-BMSCs; the activity of CD4+ T-cells was not affected without the stimulation of tumor microenvironment, which may be the cause of unimpaired inhibitory effect on the proliferation of T-cells as demonstrated previously. We found that MM- and HD-BMSCs did not show a significant apoptotic-stimulating effect. On the other hand, accumulation of senescent T-cells has been found in patients with chronic viral infections and with particular types of cancers, suggesting that different types of human cancer cells and Treg cells can induce T-cell senescence [17–20, 28]. Senescent cells, including T-cells, continue to be metabolically active but do not proliferate [19–21]. Also, the senescent T-cells show suppressor function, including impaired cytotoxicity and negative regulatory functions [20, 29, 30]. The current study showed elevated senescent naive CD4+ T-cells in BMSCs T-cells co-culture system, whereas MM-BMSCs exhibited potent senescence-inducing capacity. Herein, we found that both HD-BMSCs and MM-BMSCs induce the senescence of CD4+ T-cells; nevertheless, MM-BMSCs show a remarkable senescence-inducing effect on CD4+ T-cells. These novel findings might partially explicate the similar apoptotic rate between MM-BMSCs co-cultured T-cells and activated T-cells while the proliferative activity was downregulated by MM-BMSCs. Elevated pro-inflammatory cytokines such as IL-6 in the tumor-suppressive microenvironment and chronically infected patients may also contribute to an increased number of senescent T-cells [31]. As we observed increased level of IL-6 in MM-BMSC, we could speculate that MM-BMSCs are responsible for elevated senescent T-cells in MM microenvironment. Previous studies had shown high proportions of Th17 cells in cancer patients, and IL-17 mainly produced by Th17 contributed to tumor growth, angiogenesis, and bone destruction [32–34]. We observed that HD-BMSCs promoted the differentiation of naïve CD4+ T-cells to Treg, and the Th17-inducing effect was moderate; thus, the Treg/Th17 balance diverted to Treg. However, Th17 was overwhelming in CD4+ T-cells co-cultured with MM-BMSCs, and thus, Treg/Th17 balance shift to Th17 was consistent with elevated IL-17 and transcription factor RoRγt. As a significant relationship was demonstrated between the proportion of Th17 cells and clinical tumor stage, serum lactate dehydrogenase concentration and serum creatinine concentration, we can speculate that MM-BMSCs facilitate MM progression by Th17-inducing effect. However Th17 is involved in autoimmune diseases such as allergic asthma and rhinitis by stimulating innate immunity [35], we can conclude that MM-BMSCs in the MM microenvironment not only exert immunosuppressive effect but also evoke an immune response.

FAPα is not detectable in healthy adult tissues except remodeling tissue and healing wound [36]. Since tumor microenvironment plays a critical role in tumor growth, evasion, and immune escape, several studies demonstrated that stromal cells in tumor microenvironment expressing FAPα are associated with chronic, non-infected inflammatory lesions. Hence, the tumor growth was controlled by depleting FAP^+^ cells in an adaptive immunity-dependent manner, thereby concluding that tumor microenvironment stromal cells exert a suppressive effect on anti-tumor immunity [15, 36]. FAPα is highly expressed on the membrane of BMSCs and another kind of MSCs [11]. We hypothesized that MM-BMSCs suppress the anti-tumor immunity that is mediated by FAPα. Firstly, we identified the expression of FAPα in BMSCs from MM patients and healthy donors by immunofluorescence. However, the Western blot assay did not reveal any significant differences between the two groups. Several studies have shown that BMSCs can be educated by TCCM to convert to cancer-associated fibroblasts, and promote tumor progression [37–40]. BMSCs were separated from MM cells without interaction with MM cells. Then, we observed an elevated expression of FAPα by BMSCs treated with U266 cells-cultured medium. In addition, PT-100 reversed the negative effect of BMSCs on T-cells; both T-cells co-cultured with HD-BMSCs and MM-BMSCs regained the proliferative activity. We observed a distinct reduction in MM-BMSCs-induced senescent CD4+ T-cells when treated with PT-100. However, the effect of PT-100 on HD-BMSCs-induced senescent T-cells was moderate. We could conclude that FAPα was stimulated by MM microenvironment and mediated the immunosuppression function of MM-BMSCs. Su et al. found that tumor cells and tumor-derived fibroblasts produce a pro-inflammatory cytokine milieu, as well as, provide cell–cell contact engagement that facilitates the generation and expansion of Th17 cells [41]. IL-17 promoted the myeloma cells growth and colony formation, adhesion to BMSCs *in vitro*, as well as an increased growth *in vivo* [42]. We also observed an increase in Th17, consistent with the elevated level of transcriptional factors, RORγt and IL-17. The interplay between TGF-β and IL-6 was reported when both are expressed at high levels in MM bone marrow, which may affect the generation of Th17 cells both directly or via other pro-inflammatory cytokines, thereby modulating the antitumor immune responses [42]. TGF-β was identified as a key mediator for immune regulation. The use of neutralizing monoclonal antibodies against TGF-β1 significantly reversed the inhibition of T-cells proliferation [4, 26]. Thus, we further explored the cytokines responsible for Th17 differentiation. IL-6 and TGF-β were upregulated in the MM-BMSCs-cultured supernatant, suggesting that IL-6 and TGF-β might play a vital role in Th17 differentiation. IL-17 induces the expression of some chemokines and cytokines including IL-6 and TGF-β, in a variety of cell types, including the BMSCs [43, 44], implying that the correlation between IL-6, TGF-β, and IL-17 is responsible for the progression of myeloma. When the FAPα was inhibited, the levels of IL-6 and TGF-β were dramatically decreased. In conclusion, FAPα mediated the immunosuppression through upregulated IL-6 and TGF-β.

The PI3K pathway has since long been considered to be associated with aging; PI3K inhibitor, rapamycin, can prolong the longevity in mammals [45]. Inhibition of mTOR *in vitro* models of cellular senescence has been shown to suppress a senescent phenotype. Li et al. observed aging SLE BMSCs, which exhibited that impaired capacity of proliferation, differentiation, and secretion of cytokines was associated with the activation of PI3K/AKT/FoxO3 signaling pathway [23]. CD4+ T-cells from elderly individuals exhibited dysregulation of PI3K/NF-κB, leading to the expression of pro-inflammatory genes such as *TNF-α*, *IL-1*, *IL-6*, and *IFN-γ* that contributed to chronic inflammation associated with human aging [22]. Therefore, we proposed that the activation of PI3K pathway may implicate in BMSCs- and FAPα-induced senescence through the activation of mTOR and AKT. Furthermore, we investigated the relationship between FAPα and PI3K pathway and found that the activity of PI3K was upregulated by MM-BMSCs and downregulated by inhibiting FAPα. Elevated levels of p-AKT were found in CD4+ T-cells co-cultured with MM-BMSCs. Thus, we inhibited PI3K pathway with LY 294002, and the CD4+ T-cells restored the expression of CD28 and hTERT. Our data indicated that the involvement of PI3K/AKT pathway provided an underlying mechanism of MM-BMSCs-induced abnormalities of CD4^+^ T cells.

In conclusion, this study identified the mechanism that MM-BMSCs inhibit T-cell proliferation and drive Th17 differentiation through FAPα/TGF-β axis, leading to the progression of myeloma. Furthermore, we elucidated that FAPα-induced T-cell senescence is mediated by the PI3K signaling pathway. These findings constitute new targets for treatments by interfering with immunosuppression mediated by MM-BMSCs.

## MATERIALS AND METHODS

### BM samples

BM samples including 20 MM patients and 20 healthy donors were obtained from Wuhan Union Hospital. The characteristics of the MM patients are summarized in Table 1. All participants diagnosed first with MM did not receive any treatment. Informed consent was obtained according to procedures approved by the Ethics Committee of Wuhan Union Hospital.

**Table 1:** Characteristics of myeloma patients

Patient	Sex	Age	Clinical stage	Para protein	Karyotype	Bone lesion
1	M	49	IIA	IgG-KAP	ND	NO
2	F	55	IIIB	IgG-KAP	N	YES
3	F	54	ND	ND	N	NO
4	M	60	IIA	IgG-KAP	N	YES
5	M	61	IIIA	IgG-KAP	N	YES
6	M	64	IIA	IgG-KAP	46, XY, 1q+[2]	NO
7	M	68	IIIB	IgG-KAP	N	ND
8	F	55	IA	IgG-KAP	N	NO
9	M	53	IIIB	IgG-KAP	N	NO
10	F	62	IIIA	IgA-LAM	46, XX, 1q+, del(13)(q14)[6]	NO
11	M	50	IA	Light chain LAM	46, XY, 1q+, del(14)(q32)[6]	NO
12	M	54	IIIA	IgA-KAP	N	YES
13	F	45	IIIA	IgA-KAP	N	YES
14	F	54	IIIB	IgG-LAM	46, XX, 1q+[4]	YES
15	F	62	IIIB	IgG-LAM	N	YES
16	M	53	IA	IgD-LAM	N	NO
17	M	58	IIA	IgA-KAP	46, XY, 1q+, del(13)(q14)[4]	NO
18	M	73	IIIB	IgG-LAM	ND	NO
19	M	54	IIIB	IgG-KAP	46, XY, 1q+[4]	YES
20	M	67	IIIB	IgG-KAP	46, XY, 1q+, del(13)(q14)[6]	YES

M, male; F, female; ND, not determined; N, normal.

Clinical stage of patients was evaluated according to Durie/Salmon.

### Isolation, culture, and characterization of MSCs

BMSC have been isolated and cultured previously [3, 4]. We separated mononuclear cells from bone marrow samples obtained from 11 MM patients and 22 healthy donors, respectively, by Ficoll-Paque gradient centrifugation. The culture medium comprised of human MSCs serum-free medium containing 5% MSC-stimulatory supplements (Premedics, Beijing, China) at 37°C under 5% CO_2_. The culture medium was replaced every 3–4 days, MSCs were detached using 0.125% trypsin-0.01% EDTA, and passaged at 1:3. The passage 3 MSCs were harvested and analyzed by flow cytometry and observed positive for CD105 and CD90 and negative for CD34 and CD45 (BD, USA).

### Isolation, activation, and co-culture of CD4+ T-cells

Human peripheral blood cells from healthy donors were obtained from Wuhan blood donation center, approved by the Ethics Committee of Wuhan Union Hospital. Mononuclear cells were separated by Ficoll-Paque gradient centrifugation. The naive CD4+ T-cells were isolated from mononuclear cells using CD4+ microbeads kit (Stem Cell, Vancouver, Canada) and 95% purity was detected by flow cytometry (data not shown). The CD4+ T-cells were stimulated with the anti-CD3 functional antibody (BD). The passage 3–5 BMSCs were detached and suspended in 2 × 10^5^/mL in 12-well plates. 1 × 10^6^ CD4+ T-cells, with or without CD3 antibody stimulation, were co-cultured with 2 × 10^5^ irradiated BMSCs (30 Gy) in 12-well plates. The co-culture medium consisted of RPMI1640 supplemented with 10% FBS and 50 μg/mL IL-2 (Peprotech, Rocky Hill, USA). The co-culture of CD4+ T cells and BMSCs was set on one healthy donor to one MM patient basis.

### Immunofluorescence

BMSCs derived from MM patients and healthy donors were harvested and resuspended at a density of 1 × 10^6^ cells/mL in 6-well plates containing slides. The BMSCs were fixed in 4% formaldehyde, treated with 1% Triton X-100, incubated with rabbit anti-human FAPα polyclonal antibody (Abcam, Hong Kong, China), followed by detection using fluorescence-labeled secondary antibody. The nuclei were counterstained using 4, 6-diamidino-2-phenylindole dihydrochloride (DAPI) (Invitrogen), and images captured by Olympus B × 51.

### FAPα inhibitor

We used PT-100 (Huiqiao, Shanghai, China) to interfere the function of FAPα. PT-100 competitively inhibits the dipeptidyl peptidase (DPP) activity of FAP and CD26/DPP-IV, and a high-affinity interaction is observed with the catalytic site due to the formation of a complex between Ser630/624 and the boron of PT-100 [15]. The appropriate concentration of PT-100 was determined by T-cells and BMSCs activity cck-8 test (Supplementary Figure 2).

### Western blot

BMSCs derived from MM patients and healthy donors with or without tumor cell-cultured medium (TCCM) were lysed with RIPA buffer on ice, and the protein was quantified by the BCA protein assay kit (Tiangen Biotech Co, China), according to the manufacturer's instructions. The total protein extract was separated by 8% sodium dodecyl sulfate-polyacrylamide gel electrophoresis (SDS-PAGE) and transferred to polyvinylidene fluoride (PVDF) membranes. The membranes were blocked with 5% skim milk, incubated with 1:300 rabbit anti-human FAP polyclonal antibody (Abcam) overnight at 4°C, washed in Tris-buffered saline Tween-20 buffer (TBST), followed by incubation with HRP-conjugated goat anti-rabbit IgG (MR Biotech, China) for 1h at room temperature. The stimulated CD4+ T-cells co-cultured with BMSCs were lysed by RIPA to obtain total protein extract as described above. Primary antibodies against AKT and p-AKT (CST, Boston, USA) were used for probing the membranes. The immunoreactive protein bands were visualized by chemiluminescence (Tiangen Biotech).

### Reverse transcription-PCR analysis

BMSCs derived from MM patients and healthy donors were harvested and resuspended at a density of 1 × 10^6^cells/mL in 6-well plates. Total RNA extracted from a cell in TRIzol was used to synthesize cDNA by M-MLV Reverse Transcriptase (GeneCopoeia). The cDNA was amplified with the primers listed in Table 2, using β-actin as an endogenous control. The qRT-PCR program of FAP and β-actin was as follows: initial denaturation at 50°C for 2min and 95°C for 4min, followed by 40 cycles of 90°C for 30s and 60°C for 30s. The specificity of primers was verified by the melting curve. The 2^−ΔΔCT^ method was used to estimate the relative quantification of FAP in BMSCs. CD4+ T-lymphocytes were also processed in TRIzol as above. RORλt, Foxp3, and hTERT primer sequences are described in Table 2. The relative quantification was carried out similarly as described above.

**Table 2:** List of primers

Gene	Sequence
**FAPα**	sense 5′- TCA GCT ATG ATG CCA TTT CG-3′ antisense 5′- CCT CCC ACT TGC CAC TTG TA -3′
**FoxP3**	sense 5′- CAT TCC CAG AGT TCC TCC ACA -3′ anti-sense 5′- CAT TGA GTG TCC GCT GCT TC -3′
**RORλt**	sense 5′- GGT GGA GTT CGC CAA GAG G -3′ anti-sense 5′- CCG TGC GGT TGT CAG CAT T -3′
**hTERT**	sense 5′- TGC TAC GGC GAC ATG GAG AAC AA -3′ anti-sense 5′- TGA CAC TTC AGC CGC AAG ACC C -3′
**β-actin**	sense 5′- AGC GAG CAT CCC CCA AAG TT -3′ anti-sense 5′- GGG CAC GAA GGC TCA TCA TT -3′

### CD4+ T cell proliferation assay

1 × 10^5^ stimulated CD4+ T-cells were co-cultured with 2 × 10^4^ BMSCs derived from MM or healthy donors or cultured alone. Both were treated with or without PT-100; inactivated CD4+ T-cells were used as a control. The medium was harvested on day 6 and centrifuged at 1000 rpm to obtain T-cells, whose activity was evaluated by CCK-8 Cell Counting Kit (Dojindo, Japan), according to the manufacturer's instruction.

### Flow cytometry

1 × 10^5^ stimulated CD4+ T-cells were co-cultured with 2 × 10^4^ BMSCs derived from MM or healthy donors or cultured alone. Both cells were treated with or without PT-100; inactivated CD4+ T-cells were used as a control. CD4+ T-cells were harvested on day 3, stained with FITC-Annexin V and propidium iodide (BD, USA), and the percentage of apoptotic cells determined by flow cytometry. Other CD4+ T-cells treated similarly were harvested on day 6 and stained using FITC-CD4, PE-IL-17, Percpy5.5-CD25, and 647-Foxp3 (BD) to analyze the Treg/Th17 balance or stained by FITC-CD4 and PE-CD28 (BD). The activated CD4+ T-cells were co-cultured with BMSCs at 5:1 wherein, the PI3K pathway was inhibited with or without 25 μmol/L and 50 μmol/L LY294002, respectively. The control wells comprising of only activated CD4+ T-cells were treated with or without LY294002. The expression of CD4, IL-17, FoxP3, and CD28 by these CD4+ T-cells was also analyzed. All stained cells were assessed by FACS Calibur flow cytometer (BD) and data analyzed by FlowJo Version 7.6.1 (TreeStar).

### ELISA

Activated CD4^+^ T-cells were co-cultured with BMSCs (T-cell:BMSC = 5:1) and treated with or without PT-100. The control wells contained only the activated CD4+ T-cells. On day 6, the levels of TGF-β, IL-10, and IL-17 in supernatants were detected by ELISA (Ebioscience, USA) according to the manufacturer's instructions.

### CD4+ T-cells senescence

Stimulated CD4^+^ T-cells were either co-cultured with BMSCs at 5:1, or alone, and treated with or without PT-100; the inactivated CD4+ T-cells were used as a control. All CD4+ T-cells were harvested on day 3 and stained using SA-β-gal kit (CST, Boston, USA) to detect their senescence, which was observed blue under Olympus BX51 microscope.

### Statistical analysis

Statistical analysis was performed with the statistical SPSSv.13.0 software. The paired-sample *t*-test was used to test the probability of significant differences between samples. The results were expressed as the mean ± SEM (standard error of the mean). Statistical significance was defined as *P* < 0.05.

## SUPPLEMENTARY FIGURES


