# Supplementary material for: MM-BMSCs induce naïve CD4+ T lymphocytes dysfunction through fibroblast activation protein α
Source: Oncotarget. 2017 Apr 30;8(32):52614–28. doi: 10.18632/oncotarget.17538 (PMC5581055; doi:10.18632/oncotarget.17538)
Supplement: Supplementary file 1 [file oncotarget-08-52614-s001.pdf]

MM-BMSCs induce naïve CD4+ T lymphocytes dysfunction through fibroblast activation protein α

Supplementary Materials

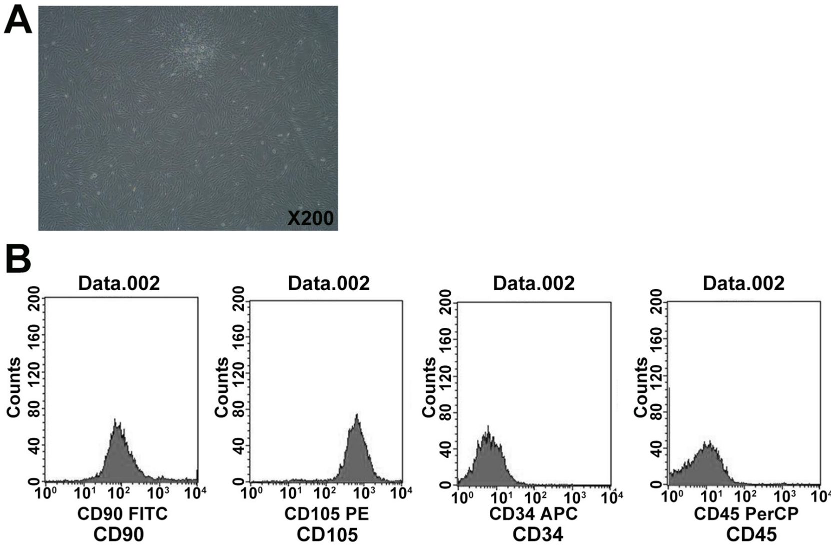

Supplementary Figure 1: The phenotype of BMSCs. (**A**) The morphological characteristics of BMSCs observed by a microscope (1:200). (**B**) The hematopoietic markers of BMSCs detected by FCM. Assays of the highest tolerant concentration of PT-100 on T-cells and BMSCs. To estimate the accurate concentration of T-cells and BMSCs, CCK-8 assay was performed. As shown in the results (Figure 1), we selected 1 pmol/mL and 0.1 pmol/mL as the accurate concentrations. At these concentrations, PT-100 did not display an obvious cytotoxicity on cells but retained its pharmacological activity.

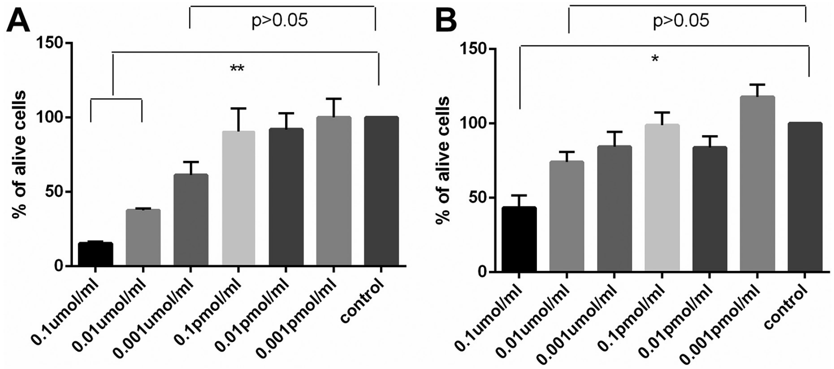

Supplementary Figure 2: The tolerant concentration of PT-100. (**A**) The activity of CD4+ T-cells and (**B**) BMSCs treated with 0.1 μmol/mL to 0.001 pmol/mL PT-100 detected by CCK-8 Kit. The OD value represented the degree of live cells, and cells without PT-100 were considered as a 100% alive ($n = 3$). $*P < 0.05$, $**P < 0.01$.
